# AI as a challenge for legal regulation – the scope of application of the artificial intelligence act proposal

**DOI:** 10.1007/s12027-022-00725-6

**Published:** 2023-01-09

**Authors:** Hannah Ruschemeier

**Affiliations:** Rechtswissenschaftliche Fakultät, FernUniversität in Hagen, Universitätsstraße 4, 58097 Hagen, Germany

**Keywords:** Artificial Intelligence, Scope of application, AIA, European Commission, Legal definitions, Regulation, Union Law

## Abstract

The proposal for the Artificial Intelligence Act is the first comprehensive attempt to legally regulate AI. Not merely because of this pioneering role, the draft has been the subject of controversial debates about whether it uses the right regulatory technique, regarding its scope of application and whether it has sufficient protective effect. Moreover, systematic questions arise as to how the regulation of constantly evolving, dynamic technologies can succeed using the means of the law. The choice of the designation as Artificial Intelligence Act leads to legal-theoretical questions of concept formation as a legal method and legislative technique. This article examines the difficulties of regulating the concept of AI using the scope of the Artificial Intelligence Act as an example.

## Introduction

The technical concept of ‘Artificial Intelligence’ (AI) seems to be the most discussed of the present day. However, it should be noted that the current hype around AI and the pursuit of technical advancement are inseparably connected to the potential for economic growth.^1^ Based on statistical algorithms which enable the process of machine and deep learning – only one aspect of the multi-layered concept of AI – great hopes are pinned on technology to improve efficiency and effectiveness and therefore society, overall economic and social welfare. AI is expected to respond to global challenges, to fight climate change, the Coronavirus and to improve public and private organisations at the micro- and macro-level. First and foremost, AI provides information and automation, for example via predictive analytics, by automating repetitive and time-consuming tasks and by analysing Big Data. AI is changing the way we work, live and even think and behave.^2^ It is also described as a disruptive technology from an economic point of view,^3^ with the potential to replace existing technologies, products or services in a fundamental way.^4^ Viewed comprehensively, AI can create risks for individuals and even whole societies. AI can affect fundamental values on which our societies are founded, leading to breaches of fundamental rights of the person, including the rights to informal self-determination,^5^ privacy and personal data protection,^6^ freedom of expression and of assembly, non-discrimination, the rights to an effective judicial remedy and a fair trial, as well as consumer protection.^7^

## Mapping AI in the legal world

It can be stated that the idea of AI is overestimated and underestimated at the same time. Overestimation is evident in the fact that expectations are being projected onto AI to solve the biggest problems of humanity without considering the human factor in the origin and solution of these problems which leads to solutionism, the belief that the development and application of the right technical feature will alone solve the problem.^8^ On the other hand, AI is underestimated, especially when it comes to the aspect of Big Data and its influence on human-built social, political and societal structures such as communication. This part of the digital transformation is irreversible and to predict its future potential is difficult because of the exponential growth of computational power alone and the interaction with the ‘analogue world’.

Eventually, this leads to the role of the rule of law and legal regulation. The functioning of law has been fundamentally challenged by ongoing technical developments and transformations for centuries. When it comes to the implications of disruptive technologies, the decision as to whether new developments demand new legal solutions, is pressing. The risks described above strengthen the arguments for general legal regulation of AI. Moreover, AI, associated with having an opaque, complex, allegedly biased and rapidly changing character does not interact well with the legal imperatives of legal certainty, transparency, explicability and equal treatment.^9^ Failures of AI which fail to meet normative expectations can cause harm, undermine trust in the institutions they use and finally hinder its development and use.^10^

The European Commission presented a unique - and so far, the first - comprehensive legal proposal for a regulation of Artificial Intelligence on 21 April 2021, the so-called Artificial Intelligence Act (or AIA). The proposal was the subject of lengthy and extensive consultations beforehand, and it is expected that the current version of the AIA will not be the final one which ultimately enters into force. By 1 June 2022, the deadline for the political groups to submit amendments to the AI Act, 3312 amendments had been submitted.^11^ The definition of AI seems to be one of the most controversial topics of the proposal, some amendments having suggested deleting the list of AI techniques and approaches found in Annex I.^12^ It is therefore all the more important to analyse the regulatory impact of the scope of application of the AIA from a legal-theoretical perspective.

Highly relevant for the practical consequences and the effectiveness of the regulation is the scope of application of the AIA. This paper examines the challenge of defining a highly dynamic regulatory subject from a theoretical perspective and the application-based perspective of the AIA. It starts with (3) the general problems of defining AI, especially considering the requirements for legal definitions followed by the (4) material and (5) territorial scope of application. The separation of powers as an important principle of the rule of law highly influences (6) how technical regulation should be designed. Consequently, the paper examines (7) the consequences of a narrow or wider scope of application within the system of the AIA itself.

## Problems with defining AI

The proposal regulates ‘AI systems’. Apart from the question of what the difference should be between ‘AI’ 13 and ‘AI systems’, the overly broad substantive scope of the AIA seems problematic.^14^

The challenges start with the fact that there is no ‘generalisable’ or fixed definition of what AI is across disciplines. The term AI is highly ambiguous, with a vast spectrum of changing definitions having been given over time.^15^

The challenge of defining AI exemplifies the difficulties of regulating technology. The regulatory goal should be oriented towards the values of fundamental rights dogmatic and the concrete protection of legal rights. However, the relevance for the protected legal interests can be justified in different ways. According to the precautionary principle, certain particularly risky products and processes, if they threaten important legal interests, are not subject to any further requirements to make them subject to legal regulation. With regard to AI, the problem arises that its effects are poorly, not assessable yet or not assessable at all. This seems to be one reason why many proposals focus so much on the technology itself and less on the impact on individuals and protected legal interests. A prognostic risk assessment according to the proportionality principle becomes correspondingly more complicated the less factual knowledge is available.

The risk profile of AI systems can therefore only be determined from an interaction between the technical functionality and the application context. From a regulatory perspective, different systems have very different risk profiles^16^ and therefore must be treated differently in relation to the protected legal interests already due to the principle of proportionality and equal treatment.^17^

### Different disciplines, different definitions

After coining the term ‘AI’ in 1956, McCarthy defined AI as ‘the science and engineering of making intelligent machines’.^18^ Or as Raymond Kurzweil points out, AI is ‘the art of creating machines that perform functions that require intelligence when performed by people.’^19^ Many subsequent definitions revolved around the aspects of acting humanly, thinking humanly, thinking rationally and acting rationally.^20^ The Encyclopaedia Britannica defines AI as ‘the ability of a digital computer or computer-controlled robot to perform tasks commonly associated with intelligent beings’.^21^ All these traditional definitions refer to human intelligence, just one indication that AI has always had strong links to psychology, cognitive science and neuroscience.^22^

Sometimes AI is equated with machine learning (ML), which is an important factor in AI but not a precise description, because machine learning is a small part of the concept of AI. ML became very popular in the last ten years inter alia due to the development of highly potent hardware, respectively Graphics Processing Unit (GPU) capacity and the availability of Big Data.^23^ Starting with the two-year doubling period of the rule of thumb known as ‘Moore’s Law’, this metric has grown by more than 300,000x since 2012 in special AI system applications.^24^ Just as important, the interaction between humans and machines has changed in a fundamental way due to the media-cultural transformations in our modern societies.^25^

In the primordial field of AI – informatics and mathematics – the term is mostly used as an umbrella term of different applications. Here, a differentiation of different kinds of AI is inevitable. Hence different AIs use different calculations and algorithms, the techniques used in AI can be roughly classified as representation, learning, rules and search.^26^

From the perspective of the humanities, AI as a term is criticised because intelligence is a natural human characteristic,^27^ it is said to be political, unprecise or even false, because AI is not comparable to human intelligence.^28^ The partly philosophical discussion about what AI could be starts with the discussion about what intelligence ‘really’ is and which ways of decision-making are plausible in a philosophical way.^29^ The concept of human intelligence and machine development have been interacting for a long time, as human intelligence is the scale mostly used to assess machine intelligence (with an AI regarded as acting intelligently, if it is able to act in a ways humans would act – or in an even better way).^30^ Artificial Neural Networks are used to model the human abilities of computing *and* learning; the major difference between the functions of neurons and the process of human reasoning is that neurons work on a sub-symbolic level. On the contrary, conscious human reasoning appears to operate at a symbolic level in the form of thoughts.^31^

### Contextual definitions and challenges

Different types of intelligence^32^ could lead to different definitions of AI even if one does not consider the technological scope. Hence intelligence from a legal point of view seems to be related to some kind of autonomy (which is another highly contested concept) resulting from the ability to adapt.^33^ As defined by the German Federal Ministry of Education and Research, AI is a branch of computer science that is focused on technical systems which are able to work on problems independently and can adapt to changing conditions.^34^ Following on from this, law currently reflects on different forms of autonomy in the field of AI (‘in the loop’, ‘on the loop’, ‘out of the loop’ and ‘post loop’),^35^$e$.$g$., in Art. 22 of the General Data Protection Regulation (GDPR). These levels are focused on the amount of human interaction during the process of AI-driven decision-making. Since neither autonomy or intelligence can be defined in a satisfactory way, context-based definitions are the only solution to legal provisions which are compliant with legal certainty and the rule of law.

After all, AI systems pose very different problems depending on who uses them, where, and for what purpose. For example, an autonomous weapon system can hardly be compared to a spam filter, even though both are based on an AI system. Indeed, this example alone illustrates the futility of lawmakers considering a general Artificial Intelligence Act that would regulate the whole phenomenon top-down, administered by an Artificial Intelligence Agency. Accordingly, it seems that there is no need for a single all-encompassing definition for ‘algorithms’ and ‘AI’.^36^ Rather, it is more important to understand the different characteristics of various algorithms and AI applications and how they are used in practice.

### Legal problems with defining AI

From a legal regulatory point of view, the definition of the regulatory subject is essential since it defines the scope of application of the regulation. But due to the broad spectrum of sciences affected either directly or indirectly by AI and societal segments, every viewpoint gives rise to its own definition of what AI is and what it means to the specific field. The fact that neither computer science or informatics are directly mentioned in the AIA shows that there is no commonly agreed technical definition of what AI is or could be. This leads to general and theoretical questions regarding requirements for legal definitions.

Based on the principles of legal certainty^37^ and the protection of legitimate expectations 38 which are both elements of the rule of law, legal definitions require inclusiveness, precision, comprehensiveness, practicability and permanence 39 in varying degrees. The inclusiveness of a legal definition has to be defined with the regard to the regulatory goal. Definitions are over-inclusive if they concern matters that are not covered by the regulatory objective and are formulated too narrowly if the protective objectives of the regulation cannot be achieved due to an excessively narrow scope of application.^40^ Precision, comprehensiveness and practicability are requirements of the rule of law principle due to the need for the principle of proportionality, legal certainty, predictability and applicability of the law.^41^ Permanence seems to be at odds with the goal of a future-proof legislation, but is rooted in the characteristic of law of creating abstract general norms for a multitude of cases of application and not to having to regulate each individual case anew.

Taken together, existing definitions of AI do not meet the most important requirements for legal definitions. They are highly over-inclusive and vague, while their understandability and practicability are debatable.^42^ The absence of a widely accepted definition thus complicates any kind of AI regulation.^43^ Additionally, the relevance of a narrower definition of the term AI is questionable, since the Act is focused on defining the risk of an AI: the fact alone that an AI fits that definition does not directly impact the risks of its use.^44^

## Material scope: definition of the AIA

The ambition of the AIA is to build a proportionate and future-proof regulatory approach.^45^ Especially when it comes to digital technology, the functioning of law is fundamentally challenged. In the first place, technology is developing in a dynamic way very fast. Law on the other hand is slow.^46^ Democracies are based on debate and compromises which take longer to negotiate – perfectly illustrated by the AIA itself. In addition, laws are passed within constitutional frameworks with certain procedural requirements. AI, associated with an opaque, complex, allegedly biased and rapidly changing character does not interact well with the legal imperatives of legal certainty, transparency, explicability and equal treatment.^47^ To avoid this conflict to begin at the first page of the AIA, the drafters avoided defining the term ‘artificial intelligence’ as such and instead laid down a very broad, but at the same time, nearly technology-neutral and therefore updatable definition of AI systems. In fact, the AIA is a ‘Software Act’.

### Definitions provided in the AIA

Article 3 (1) of the AIA defines AI systems as software that is developed with one or more of the techniques and approaches listed in Annex I and can, for a given set of human-defined objectives, generate outputs such as content, predictions, recommendations, or decisions influencing the environments they interact with.

The flexible Annex I names:

(a) Machine learning approaches, including supervised, unsupervised and reinforcement learning, using a wide variety of methods including deep learning;

(b) Logic- and knowledge-based approaches, including knowledge representation, inductive (logic) programming, knowledge bases, inference and deductive engines, (symbolic) reasoning and expert systems, and;

(c) Statistical approaches, Bayesian estimation, search and optimisation methods

As a result, the AIA in conjunction with Annex I covers almost every computer programme, merging expert systems, machine learning and statistical approaches together in a definition of AI. The one thing these have in common is that they process data.^48^ Such a broad approach may lead to legal uncertainty for developers, operators, and users of AI systems. Many associate the term ‘artificial intelligence’ primarily with machine learning, and not with simple automation processes in which pre-programmed rules are executed according to logic-based reasoning. Notwithstanding that the scope of the AIA includes every AI-system, the main regulation as regards content of the AIA is focused specifically on high-risk systems. According to recitals 1 and 5, the AI Regulation is intended, among other things, to strengthen citizens’ confidence in artificial intelligence and to promote readiness for research and innovation in the European Union. Whether this has succeeded given the very broad definition of AI procedures and approaches remains to be seen.^49^

Therefore, the material scope of the legislation is not defined by the term AI. Upon closer examination, the term is an ‘empty shell’, which the Commission may presumably have used for communications purposes. But the broad definition of AI itself must be read within the regulatory concept of the regulation. The AIA follows a risk-based approach, differentiating between three categories: forbidden, low risk and high-risk AI systems. The definition of AI itself has no filter-function for the application of the regulation. This is not a disadvantage per se, because legal methodology is largely based on conceptualisation.^50^ It is hence not the corresponding technical properties that matter, but only their impact on protected legal interests. Legally, therefore, only a context-based definition of AI is at all profitable. The goal of the AIA is to define this context-based approach via the different risk categories and these applicable risk categories are largely determined by the intended use.^51^

The classification of an AI system as high-risk is based on the intended purpose of the AI system, in line with existing product safety legislation. That is, the classification as high-risk does not only depend on the function performed by the AI system, but also on the specific purpose and modalities for which that system is used, Art. 6 AIA.

The underlying category of the intended use of an AI-systems creates legal uncertainty since it is a subjective category relating to the provider or user of the AI system. The principle of purpose limitation as a fundamental principle of the GDPR,^52^ faces a similar challenge when it comes to the processing of Big Data via predictive analytics.^53^ The challenge of regulating systems with unpredictable outcome by demanding purpose limitation for predictive analysis is not addressed by the AIA. In addition, nearly every aspect of social media use, including the moderation of content or analysis of user’s behaviour remain outside the scope of the AIA.^54^ The influence of product liability law on the systematic design can be seen very clearly in these points. The AIA appears more as a liability regime instead of formulating a protective function for affected persons and their fundamental rights. The AIA barely protects against low- and medium-risk AI.^55^

Whether an AI system is classified as high-risk or not also depends on its intended purpose, which creates a loophole for general purpose systems. Critics say that the AIA regulates specific uses of AI, but not the underlying foundation models of the application itself. 56 But general-purpose AI systems can be deployed in a variety of contexts, so if one underlying foundation model is biased, it can affect different sectors of application. The AIA in its current draft does not cover these underlying foundation models. The problem is acknowledged in the amendments to the AIA, proposed in November 2021^57^ by naming general purpose AI systems, but notwithstanding this, it is clear that they will not be automatically included within the scope of application.

The proposal fails to address the problem of narrowly defined purposes and does not appear to ensure demanding tests of necessity and proportionality.^58^ From the perspective of fundamental rights, an excessively broad definition of an AI system is not harmful *per se*. On the contrary, the goal of the AIA is to guarantee the safety of products, fundamental rights and compliance with Union Law, and the technique itself is irrelevant. It makes no difference to interferences with fundamental rights if the system which is performing facial recognition is based on ML or cryptography. But the risk categories are not defined primarily by their effects on fundamental rights rather than the social context (Annex III) and the intended purpose. Therefore, the AIA suggests a precisely defined subset of certain AI techniques but in fact covers nearly every software programme without implementing a regulatory filter for fundamental rights risks on the level of proportionality and necessity when it comes to defining the intended purpose.

### Personal scope

Article 2(1) states that the AIA applies to:

(a) providers placing on the market or putting into service AI systems in the Union, irrespective of whether those providers are established within the Union or in a third country;

(b) users of AI systems located within the Union;

(c) providers and users of AI systems that are located in a third country, where the output produced by the system is used in the Union.

Although users of AI systems are covered, the regulation focuses largely on providers, the entities that develop an AI system and either place it on the market or put it into service for their own use.^59^ From this, it follows that the AIA does not apply to private, end-users, as the definition of a user under Art. 3(4) excludes the use of AI systems in the context of a personal and non-professional activity. It also seems that research concerning AI is not included within the scope of application either.

It is noteworthy that the AIA does not provide for any rights on the part of data subjects or other persons affected by AI systems. The AIA does not acknowledge any enforcement mechanism for individuals such as procedural rights, the right to contest or seek redress or even complaint mechanisms. 60 In addition, the collective impact of AI on society as a whole is not reflected in information or participation rights of the public or of civil society groups.

## Territorial scope of the AIA

The broad area of application of the AIA directly influences its territorial scope. First and foremost, all EU member states are included, mainly through the designation of notifying authorities in Art. 30(1) and the coordination of their activities within the European Artificial Intelligence Board under Art. 53(5). Furthermore, Member States must disapply conflicting national rules and accept compliant rules on their markets.^61^ In addition, any state or corporation can theoretically be impacted by the AIA, since the regulation applies to providers placing AI on the market in the Union, users located within the Union and third-country-providers whose output is used in the Union.

The goal of the broad territorial scope of the AIA is to ensure a level playing field and effective protection of the legal interests which are addressed in the proposal. Recital 10 also mentions that the rules should apply to providers of AI systems in a non-discriminatory manner, irrespective of whether they are established or used within the Union or in a third country. Because of their ‘digital nature’, (to use the wording of recital 10), AI systems can fall within the scope of the AIA even if they are not placed on the market, put into service or used in the Union. Recital 11 describes the example of an operator established in the Union that contracts certain services to an operator outside the Union in relation to an activity.

An exception is made for public authorities of third countries because of their national sovereignty and international organisations when they are acting in the framework of international agreements concluded at national or European level for law enforcement and judicial cooperation with the Union or Member States, recital 11.

Similar to the international reach of the GDPR, Art. 3 GDPR (extra-territorial scope),^62^ the goal of the AIA is that obligations cannot be avoided, even if the provider is not situated within the EU’s jurisdiction. What matters is neither the position of the provider itself nor the user, but the position of the AI system and the place where its output is used.^63^ It is essential that the international approach of the AIA is not a territorial extension to the EU Member States (which would directly include certain states or areas), but an aterritorial approach.^64^ Therefore, there is no clear distinction between countries which are inside or outside of the EU when it comes to placing or using AI systems inside the EU. In this case, the AIA indirectly regulates foreign entities’ activities.^65^

Despite the problems of the AIA in terms of finding a more practical scope for the definition of AI, the (a)territorial scope shows a more prescient approach: not only is the European Economic Area considered, but also the global character of AI, which ensures that AI does not follow national borders.

## Separation of power

Does the principle of separation of powers necessarily require broad definitions in the area of technical regulation? Some argue, that risky technologies are best regulated by the judiciary because litigation is said to be the most cost-effective method of transferring information from litigants to lawmakers.^66^ The question is whether cost-effectiveness should be the relevant benchmark for legal regulation. In the field of regulating AI on the European level, it is crucial to create a coherent legal framework 67 in the first place to even enable the judiciary to concretise the provisions. With regard the EU and the member states, the principles of conferral and subsidiarity, Art. 5(2) and Art. 5(3) of the Treaty on the European Union (TEU), favour a functional separation of powers. A further division of sovereignty is already affected by the fact that state sovereign rights can be transferred to and exercised by a supranational holder of power. On the other hand, the transfer of sovereign rights as well as the subsequent possibilities of participating in the exercise of the sovereign rights of the European Union influences the internal structure between the powers.^68^ Hence, when it comes to effective regulation based on a European Regulation, the independencies within the multilevel system of national and European judiciary and administrational execution are crucial. Should differences of opinion among the courts not be resolved through the process of mutual understanding and agreement, the basic principle of the European Union, the idea of a democratic community of law, demands that the parliaments and citizens in the member states clarify or amend treaty law. Concerning the AIA, it is up to the Member States to establish administrative practice and case law, the interpretation of which is to be clarified by the European Court of Justice. However, in order to achieve coherent and uniform standards of protection throughout the Union, concrete specifications are indispensable in some areas at the legislative level. This is made considerably more difficult by extremely broadly formulated exceptions, such as those in Art. 5(3) and Art. 5(4) AIA, which refer to the procedural practice of the member states.

Implementation of the legal requirements of the AIA in practice will show whether a ‘No-AI-label’ will be necessary, as some critical voices have predicted on the basis of its wide scope of application.^69^ At the institutional level, it does not seem unlikely that there will be a significant shift of power from the legislative to the executive level due to the broad definitions of the AIA.

## Consequences for the regulation of AI

The AIA provides for a disproportionate role for AI providers and users in the implementation and execution of the regulation, for one thing because of the interaction between the intended purpose and the classification as high-risk systems, on the other hand because of doubtful instrument of conformity assessment. When it comes to the wording, a wide definition of ‘AI systems’ is justified in the light of the prohibited AI practices delineated in Art. 5 AIA in order to offset the threats posed by different kinds of software to the fundamental rights of individuals. Indeed, it seems to make little difference to the rights of affected citizens whether the banned practices (subliminal manipulation, exploitation of vulnerabilities, social scoring, or remote biometric identification) are enabled by machine learning or logic-based reasoning. The prohibited practices in Art. 5 nevertheless fail to protect fundamental rights of individuals due to the broad exceptions, $e$.$g$., that for biometric systems in Art. 5(2)-Art. 5(4).^70^

On the other hand, such a broad definition is too wide when it comes to high-risk AI systems. The mandatory requirements envisaged for these systems in Title III, Chap. 2 are based on the observation that a number of fundamental rights are adversely affected, in particular, by the special characteristics of ML, such as opacity, complexity, dependency on data, autonomous behaviour.^71^ Since these characteristics are either not present or only partly present in simple (logic based) algorithms, the broad definition of AI will potentially lead to overregulation. The AIA also does not clarify as to how various components of AI systems should be treated which could include either pre-trained AI systems from different manufacturers forming part of the same AI system or components of one and the very same system that are not released independently. Therefore, it should be clarified whether separate components of AI systems will be individually required to conform to the AIA and who is responsible when these components are not compliant.

The definition of AI should be connected with the level of risks the addressed systems pose for the legal interests and values the AIA wants to protect. From this point of view, the categories of risk systems in the AIA need improvement. High-risks systems listed in Annex III are categorised according to external circumstances and not according to the legal interests involved, such as ‘critical infrastructure’, ‘law enforcement’, ‘administration’ or ‘employment’. These categories arise from the presumption that there is particular relevance in these areas for the protected legal interests of the persons concerned. This may be true as a rough template, but it is far from conclusive and only rudimentarily reflects the challenges of the digital space. Questions of data protection and privacy play a role only marginally, $e$.$g$., in Art. 10(5), whereby the application of the GDPR remains unaffected. For its part, however, the GDPR has significant gaps in protection when it comes to capturing the risks posed by Big Data. The AIA does not close this gap either.

Another hiatus in the AIA can be found in excluding ‘military AI’ from the scope of the regulation. Since the regulation points out that ‘AI developed exclusively for military purposes’ (Art. 2 (3) AIA) will not be affected by the regulation, a logical and necessary appendix would define the term of exclusivity. Even if the amendments to the AIA added ‘national security purposes’ to the regions of AI system which will not be covered by the AIA, no elucidation of ‘exclusively military purposes’ can be found. In this context the AIA ignores the fact that an AI system can be used for multiple purposes (so called ‘dual use’). It is not unlikely that an AI used for civil purposes could be used in the military context and the other way around.

In addition, the AIA lacks an exception for research purposes: hence unlike Art. 89 GDPR, it makes no provision for any exception for such purposes. Therefore, in situations where researchers collaborate with industry and publish their model for academic purposes, they may run the risk of being regarded as providers who ‘develop an AI system’ with a view of ‘putting it into service’ (under Art. 3(2) AIA), $i$.$e$., supplying the system ‘for first use directly to the user or for own use’ (to use the words of Art. 3(11) AIA).^72^ This risk is magnified owing to the fact that open-source software (OSS) is an important part of the research ecosystem. Classifying any release of OSS as ‘putting into the market/into service’ and imposing conformity assessment requirements on it will simply be detrimental to the entire scientific research ecosystem.

## Footnotes

1 With an estimated market volume of €277.9 billion worldwide and a 5-year compound annual growth rate of 17.5% in 2021; *Wudel*/*Schulz* [55], p. 589.

2 *Mülhoff* [38], pp. 1868 ff.

3 *Lacy*/*Long* *et al*. [27], pp. 43-71.

4 *Danneels* [9], p. 246.

5 *André*/*Carmon et al*. [2], p. 28.

6 *Manheim*/*Kaplan* [29], pp. 106 f.

7 *Ebers*/*Hoch et al*. [11], p. 589.

8 *Morozov* [37]; *Santoni de Sio*/*Mecacci* [44] p. 1068; *Byrum*/*Benjamin* [5]. See from a legal perspective: *Mölders* [36], pp. 57 f.; *Souza* [10], pp. 1f.

9 *Ranchordas* [42], p. 89.

10 *Floridi*/*Holweg et al*. [14].

11 Draft Report on the proposal for a regulation of the European Parliament and of the Council on harmonised rules on Artificial Intelligence (Artificial Intelligence Act) and amending certain Union Legislative Acts (COM2021/0206 – C9-0146/2021 – 2021/0106(COD), 2021/0106(COD). 226 of those amendments refer to Art. 3 AIA, the article which includes the legal definition of AI systems.

12 Draft Report on the proposal for a regulation of the European Parliament and of the Council on harmonised rules on Artificial Intelligence (Artificial Intelligence Act) and amending certain Union Legislative Acts (COM2021/0206 – C9-0146/2021 – 2021/0106(COD), 2021/0106(COD).

13 See p. 11; recitals (28), (38), (39) (40), and (47) AIA.

14 See p. 11; recitals (28), (38), (39) (40), and (47) AIA.

15 *Schuett* [46], p. 4; *Wang* [53], pp. 7f.

16 Whereas apps and web services in the medical field are more likely to carry risks concerning data security, an AI system used in criminal prosecutions may carry the risk of being discriminatory.

17 As important factors of the rule of law; *Huscroft*/*Miller* [22], pp. 1 f.

18 *McCarthy* [32], p. 2.

19 *Kurzweil* [26], p. 14

20 *Russell*/*Norvig* [43], pp. 20-22.

21 *Copeland* [8].

22 *Chowdhar*y [6], pp. 7 f.; *Ertel* [13], pp. 4 f.

23 Data volume has risen at a higher rate than processing speed which requires new techniques to close the gap since a computer is only as fast as its weakest part. Therefore, parallel processing is necessary. *Parhami* [41], p. 1253.

24 https://openai.com/blog/ai-and-compute/.

25 *Mühlhoff* [38], p. 1870.

26 *Chowdhary* [6], p. 9.

27 *Wang* [53], p. 8 on different abstractions of human intelligence.

28 *Korteling*/*van de Boer-Visschedijk* et al. [25].

29 As an example, an AI which should make a decision on the basis of a deontological set or moral would mostly take different decisions to those of an AI which is based on a consequentialist set, *Misselhorn* [33], p. 191.

30 *Misselhorn* [33], p. 17.

31 *Chowdhary* [6], p. 8.

32 *Gardner* [16], pp. 13-34.

33 *Sternberg* [48], develops a theory of adaptive intelligence.

34 Bundesministerium für Bildung und Forschung, Sachstand Künstliche Intelligenz 2019, p. 1.

35 Although, the humans in the loop can be poorly equipped to review the suggestions of an algorithm: *Mittelstadt*/*Allo et al*. [34], p. 11. From a medical point of view: *Jotterand*/*Bosco* [23], p. 2455.

36 *Wang* [53], p. 17 proposes the working definition “Intelligence is the capacity of an information”.

37 *Blanke* [3], pp. 89 f.

38 On the concept of legitimate expectations: *Forsyth* [15], pp. 238 f.; https://www.bverwg.de/medien/pdf/rede_20160421_vilnius_rennert_en.pdf.

39 *Schuett* [45]. Empirical study on how experts and non-experts interpret legal provisions: *Gordon* [19], p. 273.

40 See *Schuett* [46], p. 1 for an overview of the categories and their implications.

41 *Tamanaha* [51], pp. 131 f. For the German Rechtsstaatsprinzip, $e$.$g$., *Scholz* [31], recital. 76.

42 Schuett [46], p. 1.

43 *Glauner* [17] p. 3.

44 *Svantesson* [51], p. 2.

45 Proposal for a Regulation of the European Parliament and of the Council laying down harmonised Rules on Artificial Intelligence (Artificial Intelligence Act) and amending certain legislative Acts, COM/2021/206 final.

46 *Hoffmann-Riem* [20], p. 14.

47 *Ranchordas* [42], p. 89.

48 *Mökander*/*Axente et al*. [35], p. 18.

49 *Engelmann*/*Brunotte et al*. [12], p. 318.

50 *Larenz* [28], p. 412.

51 See Art. 3(12); Art. 6a), Art. 7(2 a).

52 See Art. 5(1)(b) GDPR

53 *Mühlhoff* [39].

54 *Stuurman*/*Lachaud* [49]. See *Greenleaf* [20] on how the AIA affects the processing of personal data.

55 *Stuurman*/*Lachaud* [49].

56 https://sciencebusiness.net/news/new-type-powerful-artificial-intelligence-could-make-eus-new-law-obsolete.

57 Draft Report on the proposal for a regulation of the European Parliament and of the Council on harmonised rules on Artificial Intelligence (Artificial Intelligence Act) and amending certain Union Legislative Acts (COM2021/0206 – C9-0146/2021 – 2021/0106(COD), 2021/0106(COD).

58 *Smuha*/*Ahmed-Rengers et al*. [47].

59 *McCarthy* [32], p. 2.

60 *Smuha*/*Ahmed-Rengers et al*. [47].

61 *Veale*/*Borgesius* [52], p. 97.

62 $E$.$g$., *Ernst* [40], recital. 13; *Gömann* [18], p. 567.

63 *Bomhard*/*Merkle* [4], p. 279.

64 *Floridi et al*. [14], p. 216.

65 *Svantesson* [50], p. 5.

66 *Kołacz*/*Quintavalla et al*. [24], p. 21 who distinguish between risky and uncertain technologies, while the risk potential of the latter is unpredictable.

67 On the question of unified dogmatics of fundamental rights on a european level:*Classen* [7], p. 279.

68 *Grzeszick* [30], recital. 114.

69 Svantesson [50], p. 5.

70 *Ebers*/*Hoch et al*. [11], p. 590.

71 See p. 11 at 3.5.

72 See p. 16.
